# A Secondary Analysis of Maternal Ultra-processed Food Intake in Women with Overweight Or Obesity and Associations with Gestational Weight Gain and Neonatal Body Composition Outcomes

**DOI:** 10.34763/jmotherandchild.20212504.d-21-00025

**Published:** 2022-03-23

**Authors:** Kathryn Whyte, Isobel Contento, Randi Wolf, Laura Guerra, Euridice Martinez, Xavier Pi-Sunyer, Dympna Gallagher

**Affiliations:** 1 New York Nutrition Obesity Research Center, Division of Endocrinology, Department of Medicine, Columbia University Irving Medical Center, New York, New York, United States of America; 2 Program of Nutrition, Department of Health and Behavior Studies, Teachers College Columbia University New York, New York, New York United States of America; 3 Department of Nutrition, School of Public Health, University of Sao Paolo, Sao Paolo, Brazil; 4 Institute of Human Nutrition, College of Physicians and Surgeons, Columbia University New York, New York, United States of America

**Keywords:** neonatal body composition, quantitative magnetic resonance, ultra-processed food, gestational weight gain

## Abstract

**Background:**

This study is an observational secondary analysis of the Lifestyle Intervention for Two (LIFT) randomised controlled trial data. There is a paucity of data related to mechanisms of health effects and dietary intake of ultra-processed foods (UPF). Earlier studies demonstrate associations between greater UPF intake and weight gain. The purpose of the study was to describe associations among maternal UPF intake with gestational weight gain (GWG) and neonatal body composition.

**Material and methods:**

Women with overweight or obesity (n=156) and offspring (n=126) with complete energy intake, anthropometrics and body composition measures were selected. Maternal weights and diet recalls (Automated Self-Administered 24) were measured at weeks 14 and 35 gestational age (GA). Body composition was assessed by infant quantitative magnetic resonance (infant-QMR) and air displacement plethysmography (ADP) at birth. Dependent variables were GWG and neonatal fat mass, fat-free mass, and lean mass at birth; covariates were dietary, socioeconomic and biological. Stepwise linear regressions were used to test associations.

**Results:**

Highest quartile of percentage of energy intake from UPF (PEI-UPF) was not significantly correlated with maternal GWG (p=0.215), infant QMR fat (p=0.816) and lean mass (p=0.423) or ADP fat (p=0.482) or fat-free mass (p=0.835).

**Conclusions:**

While no significant associations with UPF were observed in this smaller size cohort, further investigations would be justified in larger cohorts on the relationships of maternal UPF intake and GWG and offspring outcomes. Clinical Trial NCT01616147

## Introduction

Obesity in children and adults is associated with shorter life expectancy and multiple co-morbidities [1]. One-third of pregnant women experience excessive gestational weight gain (GWG) [2]. Excessive GWG is a strong predictor of postpartum weight retention, which can contribute to obesity in women of childbearing age [2]. One US study estimated the annual cost of maternal overweight, gestational diabetes mellitus (GDM), and related macrosomia during the perinatal period to exceed $1.8 billion, not including the long-term consequences to offspring health [3]. Accordingly, the economic burden of maternal overweight and related comorbidities is significant. Nevertheless, while obesity is widely recognised as a global issue, developing obesity because of excessive GWG in women of child-bearing age is often overlooked [4]. Consequently, there is a paucity of information and best-practice strategies related to weight management during pregnancy.

Maternal diet during pregnancy has the potential to impact fetal development and influence future metabolic disease risk [5, 6, 7]. Excess calorie consumption, dietary fat content and micronutrients (e.g. vitamins D, B12) play an important role in fetal programming, but specific intrauterine mechanisms remain poorly understood [8]. Little is known about when and how specific nutrient exposures impact human fetal programming – specifically fetal fat accretion [9, 10, 11, 12]. While maternal dietary fat is an obvious contributor to the lipid substrate for fetal adipose tissue growth, the micronutrient components of the diet may also play a role in directly modulating cellular mechanisms responsible for adipogenesis [13, 14]. There is no consensus on the most-effective content, format or theoretical framework for GWG lifestyle interventions [2, 15]. The existing literature on the effects of lifestyle interventions to promote healthy GWG via behavioural strategies during pregnancy reveal mixed results with major issues related to adherence, efficacy and feasibility posing significant limitations [15, 16, 17, 18, 19].

Recently, the literature has demonstrated an increasing share of ultra-processed foods (UPFs) in the average American diet [20] and associations of UPF intake with suboptimal health outcomes [21, 22, 23, 24, 25, 26, 27]. There are currently no dietary recommendations around UPFs nor does the Healthy Eating Index (HEI), a measure of diet quality, address food processing. The NOVA [27,28] approach proposes that foods can be categorised into the following groups: 1. unprocessed or minimally processed foods, 2. processed culinary ingredients, 3. processed foods and 4. UPF. UPF, according to the NOVA classification, are industrial formulations of processed food substances (oils, fats, sugars and starch, often chemically modified) contain little or no whole food and typically include flavorings, colorings, emulsifiers and other cosmetic additives [28]. Studies that examined the dietary share of percentage of energy intake (PEI) from UPFs at the national level and in several countries report significant associations between UPF consumption and obesity, non-communicable diseases (NCDs), and other metabolic perturbations in populations throughout the life cycle [21, 22, 23,25, 26, 27,29]. A study by Rohatgi et al. [30] assessed the relationship between maternal diet quality by NOVA and GWG and neonatal body composition as measured by skinfold. The investigators observed in a sample of n=45 women with normal weight and obesity that a diet higher in UPF predicted greater offspring fat mass (FM) by skinfold measurements [30]. In this study, a 1%-point increase in in percentage of energy intake of UPF (PEI-UPF) was significantly associated with a 1.33 kg increase in GWG and 0.62 percentage points in neonatal total adiposity [30].

The Lifestyle Intervention for Two (LIFT) trial [18] is a behavioural lifestyle intervention randomized controlled trial (RCT) conducted during the 2nd and 3rd trimesters in n=210 women with overweight or obesity. The LIFT trial did not find associations of improved maternal diet quality as measured by the HEI with the observed difference of greater lean mass (LM) in the intervention group offspring [18]. The current study is a secondary analysis of the LIFT trial data to test the hypothesis that maternal UPF intake as measured by the NOVA classification is significantly associated with neonatal body composition (g) and maternal GWG (kg).

## Material and methods

### Participants and recruitment

**Figure 1:**
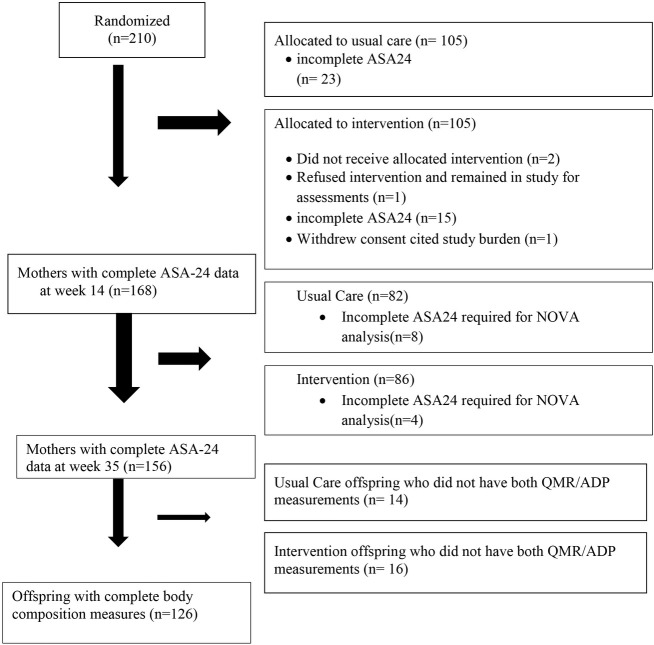
Enrollment and Analysis

Dietary data for this analysis came from the LIFT trial [18], a product of the LIFEMOMS Consortium [4,31], which was a randomised controlled trial of a behavioural lifestyle intervention (delivered by behavioural lifestyle interventionists including registered dietetic nutritionists) delivered to women with pre-gravid overweight or obesity between week 14 and week 35 gestation [18]. The parent study was approved by and conducted in accordance with the Institutional Review Boards at St Luke’s-Roosevelt Hospital and Columbia University Irving Medical Center (IRB-AAAO0651). Study participants provided written informed consent prior to participation. The intervention program was derived from the Diabetes Prevention Program and the Action for Health in Diabetes (Look AHEAD) curricula with the focus modified from weight loss to GWG control as recommended by the Institute of Medicine (IOM) guidelines [18]. Maternal baseline characteristics by intervention and usual care groups are presented in Supplementary Table 1. Eligibility criteria included age >18 years; body mass index (BMI) >25 at baseline measurement and a singleton pregnancy. Women with diabetes mellitus or GDM as evidenced by a glycosylated hemoglobin greater than 6.5% at study screening were excluded. Gestational age (GA) was confirmed by ultrasound [18]. Women with complete maternal dietary data (n=156) and their offspring having both QMR and ADP measures (n=126) were included in the analyses (Figure 1).

## Measures

Maternal dietary data were collected using the Automated Self-Administered 24-hour (ASA-24) recall, administered to study participants at weeks 14 and 35 of pregnancy. Data were delimited to participants who completed ASA-24 recalls both pre-intervention (week 14) and post-intervention (week 35) and where the recall was visually checked for completeness by study personnel and reported by the participant as being representative of typical intake. From the ASA-24, we classified all recorded food items according to NOVA. Nine-digit food codes as provided by the ASA-24 [32] or their underlying ingredient codes (SR or standard reference codes, Release 22) for handmade recipes used to code food intake were assigned to one of the four mutually exclusive NOVA groups. The energy content and weight of foods reported in the ASA-24 were derived using the FNDDS 4.1 and the SR release 22 and used to estimate the PEI of each NOVA group per day. The average week 14 and week 35 energy contribution of UPF was divided into quartiles of UPF intake with n=39 participants per quartile and used as our main exposure variable. Quartiles were graphed and visually checked to be normally distributed. The first quartile (Q1) included the lowest consumers of UPF with average PEI-UPF between 0 and 36.09%. The second quartile (Q2) included those with PEI-UPF between 36.10 and 45.23%. The third quartile (Q3) included those with PEI-UPF between 45.24 and 54.73%. Finally, the fourth quartile (Q4) included the highest consumers of UPF with PEI-UPF between 54.74 and 75.25%.

Food intake data were also analysed using the HEI [33]. The HEI is a diet quality index that measures conformance with federal dietary guidance. The HEI-2010 includes 12 components, nine of which assess adequacy of the diet including (a) total fruit, (b) whole fruit, (c) total vegetables, (d) greens and beans, (e) whole grains, (f) dairy, (g) total protein foods, (h) seafood and plant proteins and (i) fatty acids. The remaining three—refined grains, sodium, and empty calories—assess dietary components that should be consumed in moderation. For all components, higher scores reflect better diet quality.

STATA SE 14 was used for the assignment of NOVA categories and HEI scores to ASA24 data (**Stata** Statistical Software: Release **14**. College Station, TX: StataCorp LP. StataCorp, 2014). The NOVA categories and HEI score variables were saved in the dataset.

Maternal weight was measured to the nearest 0.1 kg using a Tanita scale (BWB-800, Tanita Corp., Arlington Heights, Illinois). Maternal weight gain was defined as the difference between the study-measured weight at week 35 (post-intervention) and week 14 weight (pre-intervention). Week 14 weight was measured at or before randomisation and no later than 15 weeks and 6 days GA [18]. Week 35 weight was measured between 35 weeks and 36 weeks and 6 days [18]. No differences in UPF intake from the ASA-24 were found between intervention and usual care groups. Accordingly, the groups were pooled for all subsequent analyses. Maternal ASA-24 and GWG differences between intervention and control groups are described in Supplementary Table 2.

Neonate birth characteristics (unadjusted) by intervention/control groups are described in Supplementary Table 3. Neonatal body composition at 1–3 days after birth was measured using two independent methods—air displacement plethysmography (ADP) and quantitative magnetic resonance (QMR). The PEA POD Infant Body Composition System (COSMED USA Inc., Concord, California) is an infant-sized ADP system that measures infant body weight and estimates body volume from which FM and fat-free mass (FFM) are derived [34,35]. This system has been validated in infants [34]. In a separate validation study, repeated measures on the same day in 29 infants gave CVs of 6.5% for FM and 1.1% for FFM [35].

The QMR is a non-imaging technique (EchoMRI-Infants^TM^) that uses an electromagnetic field to detect the hydrogen atoms of FM, LM and total body water (TBW) [36]. Once excited by radiofrequency pulses, these protons have different relaxation times relative to the tissue (fat) or medium (water) in which they are embedded. The processed signal is obtained from the whole body at once. The QMR accommodated children up to 12 kg, which corresponds to approximately 12 months of age. In a separate validation study, repeated measures on the same day in 14 newborns measured three times showed high precision, 5.3% for FM, 2.5% for LM and 1.6% for TBW [37]. While ADP is a well-established measurement method for body composition in infants under 8 kg, QMR allows for the measurement of the two main body composition compartments (fat and LM) in children up to 12 kg and provides assessment of hydration.

## Data analysis

Maternal and offspring sociodemographic characteristics and maternal diet quality were described according to quartiles of dietary contributions of UPF for n=156 women. ANOVA-tests were used to compare mean values and chi-square and Fisher’s tests to compare frequencies across the quartiles of UPF consumption. Week 14 and week 35 mean differences of NOVA groups energy contributions, total kilocalorie intake and HEI scores, both overall and in each quartile of PEI-UPF (mean week 14 and week 35) were assessed using t-tests. Pearson and point biserial correlations were used to correlate gestational weight gain and neonatal body composition with covariates, to identify potential confounders and effect modifiers for regression models. These included average total calorie intake (kcal) and HEI-2010 score (0–100), gestational age (week), age (years), baseline BMI (continuous), parity (1, 2 or 3+), race (white or non-white), income (below $75,000 or ≥ $75,000), education (college degree yes/no), group (usual care/ intervention); offspring covariates included gestational age (days), sex and length (cm). Ordinary least squares linear regressions assessed the relationship between quartiles of UPF consumption and GWG and neonatal body composition. Assumptions supporting the use of linear regression analysis related to independence, normality, homoscedasticity and outliers were tested, and no violations were found. The predictors and interactions were examined in a series of models. The investigation began with a full model of all significantly associated covariates. Covariates that were not significant in the regression models were withdrawn from the model if no change in the quartile coefficients were observed. The final parsimonious model is presented. These analyses were conducted in STATA/IC 16.1 (**Stata** Statistical Software: Release **16**. College Station, TX: StataCorp LP. StataCorp, 2019) was used for the analyses.

## Results

Table 1 presents maternal and offspring descriptive characteristics according to quartiles of PEI-UPF intake. Maternal baseline descriptive characteristics did not differ across the quartiles. As expected, NOVA groups 1–3 (unprocessed, culinary ingredients and processed groups) energy contributions monotonically and significantly decreased across the quartiles of UPF intake while NOVA group 4 increased. Average HEI scores also decreased significantly across quartiles while no change was observed in total energy intake (kcals) (p<0.01). There were no differences between quartiles relative to offspring covariates, except sex (p=0.04) (Table 1).

**Table 1:** Maternal and offspring descriptive analysis by quartiles of percentage of energy intake of ultra-processed foods (PEI-UPF).

Maternal^+^	PEI-UPF Quartiles				*p*
	Q1 (0-36.09%) N=39 Mean+SD	Q2 (36.10-45.23%) N=39 Mean+SD	Q3 (45.24-54.73%) N=39 Mean+SD	Q4 (54.74-75.25%) N=39 Mean+SD	
%G1^a^	51.5±14.0	42.8±17.5	36.2±11.8	28.4±13	**<0.01**
%G2^a^	6.5±6.8	5.8±7.8	4.9±5.5	3.6±4.7	**0.04**
%G3^a^	13.1±12.2	10.7±9.7	9.4±9.6	6.9±5.8	**0.03**
%G4^a^	28.8±14.8	40.7±14.0	49.6±13.3	61.2±16.5	**<0.01**
Total kcals^a^	1875.9±926.2	1863.6±1222.7	1953±886.8	1921.8±807.5	0.78
HEI 0-100^a^	62.7±12	59.2±15.4	56.1±8.3	52.9±11.9	**<0.01**
Gestational Age (week)^a^	14.5±0.72	14.6±0.59	14.4±0.80	14.2±0.84	0.17
Maternal Age (years)^a^	34.1±3.9	33.7±4.5	33.5±3.2	32.5±4.8	0.35
Baseline BMI (kg/m^2^)^a^	29.8±4.0	30.2±4.0	31.2±5.8	30.3±4.9	0.33
Parity (%)^b^					0.45
1	25.6	43.6	30.8	33.3	
2	33.3	30.8	25.6	38.5	
3+	41.1	25.6	43.6	28.2	
Race (%)^b^					0.68
White	38.5	51.3	40.0	46.2	
Non-white	61.5	48.7	60.0	53.8	
Income (%)^b^					0.96
≤$75,000	33.3	33.3	35.9	38.5	
>$75,000	66.7	66.7	64.1	61.4	
College (%)^b^					0.12
No	23.1	12.8	25.6	7.7	
Yes	76.9	87.2	74.4	92.3	
Group (%)^b^					0.77
Usual Care	40.0	51.3	46.2	51.3	
Lifestyle Intervention	60.0	48.7	53.8	48.7	
**Offspring^++^**	**Q1 N=32**	**Q2 N=31**	**Q3 N=32**	**Q4 N=31**	**p**
Length (cm) (mean±SD)^a^	49.8±1.9	49.85±2.0	50.08±1.8	49.55±2.3	0.79
Age (days) (mean±SD)^a^	2.75±2.1	2.62±2.0	2.16±1.8	2.45±1.9	0.65
Sex (%)^b^					**0.04**
Female	15 (46.9)	21 (67.7)	12 (37.5)	11 (35.5)	
Male	17 (53.1)	10 (32.3)	20 (62.5)	20 (64.5)	
Race (%)^b^					0.76
White	59.4	51.6	62.5	51.6	
Non-white	40.6	49.4	37.5	49.4	

a = one-way ANOVA, ^b^= chi square *Statistically significant mean difference between quartiles: p<0.05 ^+^Maternal Quartiles (mean week 14 and 35) (n=156) (mean, min-max range): Q1 =28.8 (12.8-36.09%), Q2=40.7 (36.10-45.23%), Q3=49.6 (45.24-54.73%), Q4=61.2 (54.74-75.25%); ^++^Neonatal Quartiles (n=126) (mean week 14 and 25, min-max range): Q1=31 (13.5-36.4%, Q2=32(36.6-45.96%), Q3=32(45.97-54.78%), Q4=31(54.79-75.25%) G1 = unprocessed foods, G2= minimally processed culinary ingredients, G3= processed foods, G4= ultra-processed foods; %= contribution to total energy intake; kcals=kilocalorie; HEI=Healthy Eating Index

In Table 2, associations between maternal and neonatal covariates and the outcomes of GWG, QMR-LM, QMR-FM, QMR-TBW, ADP-FFM and ADP-FM were investigated for significant covariation to determine potential confounders and effect-modifiers in the association between PEI-UPF and these outcomes. GWG was significantly greater in women who were white, in usual care (UC) and had university degrees and with lower baseline BMIs and HEI scores. QMR-LM was significantly greater in offspring who were male, in the lifestyle intervention (LI) group and were of greater length at study measure. QMR-FM was significantly greater in offspring with mothers who did not have university degrees who were male and of greater length at study measure. QMR-TBW was significantly greater in offspring who were male and of greater length at study measure. ADP-FFM was significantly greater in offspring who were male, were non-white and of greater length at study measure. ADP-FM was significantly greater in offspring born to mothers who were younger, with greater baseline BMIs and did not hold university degrees and were female and with greater length at study measure.

**Table 2:** Maternal and offspring correlational analysis of gestational weight gain and neonatal body composition outcome variables with potential covariates.

Maternal^+^	Gestational Weight Gain	Lean Mass (QMR)	Fat Mass (QMR)	Total Body Water (QMR)	Fat Free Mass (ADP)	Fat Mass (ADP)
Total Kcals	0.05	-0.01	0.03	0.03	0.02	0.03
HEI Average Score 0-100 ^a^	**0.14**	-0.03	-0.03	-0.06	-0.11	0.02
Gestational Age (week)^a^	-0.004	-0.10	-0.03	-0.07	-0.07	0.02
Maternal Age (years)^a^	-0.06	-0.16	-0.12	-0.20	-0.16	**-0.17**
Baseline BMI (kg/m^2^)^a^	**-0.31**	0.11	0.17	0.12	0.10	**0.22**
Parity^b^	-0.15	-0.06	0.12	0.03	-0.05	0.14
Race^c^	**-0.24**	0.12	0.02	0.07	**0.17**	-0.14
Income^c^	0.03	0.04	-0.12	-0.02	0.006	-0.13
College^c^	**0.17**	-0.08	**-0.20**	-0.14	-0.07	**-0.24**
Group^c^	**-0.21**	**0.19**	0.06	-0.16	-0.12	-0.08
**Offspring^++^**						
Length^a^ (cm)	--	**0.79**	**0.57**	**0.76**	**0.81**	**0.44**
Age^a^ (years)	**--**	-0.03	-0.07	-0.01	0.02	-0.09
Sex^c^	--	**-0.32**	**-0.32**	**-0.36**	**-0.32**	**0.24**

a =Pearson correlation; ^b^=partial correlation ^c^=biserial correlation Bold type: Statistically significant correlation =p<0.05 BMI: body mass index; HEI: Healthy Eating Index, Parity: 1, 2, 3+; Race: white or nonwhite Income: <$75,000, >$75,000; College: yes, no; Group: lifestyle intervention or usual care; Obesity: with overweight but no obesity, obesity; Sex: male or female

As shown in Table 3, linear regression was used to examine the effects of PEI-UPF quartiles on GWG. Marginal means and the 95% confidence intervals for unadjusted and adjusted models are presented. For the adjusted model, covariates included group assignment and having obesity at baseline. PEI-UPF quartiles were not significantly associated with GWG. Again, using linear regression, maternal PEI-UPF quartiles were not significantly associated with offspring body composition outcome variables in either unadjusted or adjusted models (Table 3).

**Table 3:** Marginal means from ordinary least squares regressions of gestational weight gain (n=156) and neonatal body composition variables (n=126) according to quartiles of percentage of energy intake of ultra-processed foods (PEI-UPF)

	PEI-UPF^1^ Q1			PEI-UPF^1^ Q2			PEI-UPF^1^ Q3			PEI-UPF^1^ Q4			*Ptrend*
	Marginal Means	95% CI		Marginal Means	95% CI		Marginal Means	95% CI		Marginal Means	95% CI		
**Maternal**													
**GWG (kg)**													
Unadjusted	8.84	7.5	10.2	10.05	8.7	11.4	8.26	6.9	9.6	8.34	7.0	9.7	0.213
Adjusted^a2^	8.82	7.2	10.4	10.09	8.5	11.7	8.33	6.7	9.9	8.26	6.7	9.8	0.215
**Neonatal**													
**Lean Mass (g)**													
Unadjusted	2.30	2.2	2.4	2.28	2.2	2.4	2.38	2.3	2.5	2.29	2.2	2.4	0.414
Adjusted^a3^	2.30	2.2	2.4	2.30	2.2	2.4	2.34	2.3	2.4	2.32	2.3	2.4	0.423
**Fat Mass(g)**													
Unadjusted	.558	.50	.62	.559	.50	.62	.568	.51	.62	.503	.44	.56	0.224
Adjusted^a4^	.554	.48	.63	.544	.47	.62	.569	.50	.64	.521	.45	.60	0.816
**TBW(g)**													
Unadjusted	2.42	2.3	2.5	2.39	2.3	2.5	2.52	2.4	2.6	2.41	2.3	2.5	0.457
Adjusted^a5^	2.42	2.3	2.5	2.41	2.3	2.5	2.49	2.4	2.6	2.43	2.3	2.5	0.573
**FFM(g)**													
Unadjusted	2.89	2.8	3.0	2.81	2.7	2.9	2.96	2.8	3.1	2.85	2.7	3.0	0.568
Adjusted^a6^	2.90	2.8	3.0	2.82	2.7	2.9	2.92	2.8	3.0	2.88	2.8	3.0	0.835
**Fat Mass (g)**													
Unadjusted	.353	.30	.41	.366	.31	.42	.357	.30	.41	.294	.24	.35	0.119
Adjusted^a7^	.350	.29	.41	.340	.28	.40	.351	.29	.41	.327	.26	.39	0.482

a Model marginal means and confidence intervals presented with Bonferroni correction ^1^Maternal Quartiles (n=156) (mean, min-max range) 45.1% (12.8-75.25): Q1 =39 (12.8-36.09%), Q2=39 (36.10-45.23%), Q3=39 (45.24-54.73%), Q4=39 (54.74-75.25%); Neonatal Quartiles (n=126) (mean, min-max range): Q1=31 (13.5-36.4%, Q2=32(36.6-45.96%), Q3=32(45.97-54.78%), Q4=31(54.79-75.25%) ^2^Gestational Weight Gain (GWG) adjusted for group assignment (reference is Usual Care) and obesity (reference is with overweight but not obesity) ^3^Lean Mass adjusted for sex (reference is male), length (cm) and group (reference is Usual Care) ^4^Fat Mass (QMR) adjusted for college (reference is no) length (cm) and sex (reference is male) ^5^Total Body Water (TBW) adjusted for length (cm) and sex (reference is male) ^6^Fat Free Mass (FFM) adjusted for length (cm) and sex (reference is male) ^7^Fat Mass (ADP) adjusted for college (reference is no) length (cm) sex (reference is male

## Discussion

This study sought to examine the associations between diet quality during pregnancy, specifically of percent UPF consumed as measured by the NOVA classification, and maternal excessive GWG and neonatal body composition. The current analysis found no significant associations between quartiles of PEI-UPF and GWG or offspring body composition. This secondary analysis replicates the findings from the primary LIFT study that maternal diet, as measured by HEI, was not related to observed differences between offspring [18], despite the intervention successfully mitigating excessive GWG and having a measurable impact on neonatal body composition.

Epidemiological studies have linked greater UPF intake to various adverse health outcomes [22, 23, 24, 25, 26]. The lack of significant correlation between UPF intake and GWG or neonatal body composition in this study may be due to several factors including the intervention or the average cohort intake of UPF.

First, the intervention in the parent study was successful at controlling GWG within IOM guidelines, which may have diluted the association between PEI-UPF and GWG. As shown in Supplementary Table 4, which compares women who gained more or less than the statistical mode of GWG for this cohort (7.4 kg), there was a significant difference between women in the LI group compared to women in the UC group. The statistical mode of GWG was used in place of traditional IOM excessive GWG guidelines due to the mitigating effect on GWG of the intervention. Of the women who gained more than the mode, 45% were in the LI, compared to the 64% in LI who gained less than the mode. Additionally, the fact that women in Q4 PEI-UPF had significant improvement in HEI score between week 14 and week 35 may also be related to the success of the intervention in attenuating GWG, which could be ascribed to an improvement of the nutrient profile of consumed foods after the intervention even within high UPF consumers.

Another factor contributing to the observed lack of association between UPF and GWG may be related to the current cohort average intake of 39.7% unprocessed foods, which is well above the national average of 30% [20]. This may be due to increased access to unprocessed foods; as shown in Table 1, the majority of women in this study reported an annual income ≥$75,000. The intervention was delivered with messages to reduce calories from sources of fat. As there were no differences between quartiles PEI-UPF in total calories, it is plausible that women with higher UPF intakes chose foods that were marketed as low calorie, allowing for GWG attenuation. There are no mechanistic studies to explain the metabolic pathways associated with UPF intake and weight gain; it is unknown if lower calorie UPFs incorporated in a diet that meets energy requirements are associated with adverse health outcomes. One RCT observed significant weight gain during an 81% UPF intake phase [38]. In that study, outcomes related to energy expenditure and metabolic health differed significantly in a cross over repeated measurements design under conditions of unprocessed dietary intake and ultra-processed dietary intake. During the ultra-processed dietary intake phase, participants consumed more of the provided calories and gained more weight despite being presented with matched calorie diets. Moreover, participants had higher respiratory quotients, measured by a 24-hr respiratory chamber on the UPF diet [38]. In the context of the current cohort with a higher than national average baseline for unprocessed foods and lower than national average UPF intake (well below 81% as presented in that study) and in the presence of a lifestyle intervention RCT, observation of effect size excessive weight gain may not occur. This would require further investigation into more precise guidelines surrounding UPF intake.

The current study findings differ from those of a study by Rohatgi et al. [30] that assessed the relationship between maternal diet quality by NOVA and GWG and neonatal body composition. The investigators observed that a diet higher in UPF predicted greater offspring FM by skinfold measurements [30]. There are several methodological differences between that study and the current one that make comparisons difficult, including overall study design (an observational cohort study versus an intervention), use of a food frequency questionnaire for maternal dietary intakes, and skinfolds as measures of neonatal FM. Most importantly, the mean PEI-UPF intake for the cohort of n=45 women with lean or with obesity was 54.4±13.2%, compared to the current study’s cohort of women with overweight or obesity of 45.1±12.7%. Furthermore, the mean for the Rohatgi study cohort would be categorised in Q4 PEI-UPF, the highest quartile, in the current study. Additionally, the sample in the Rohatgi study of n=45 women were with lean (35.6%) and obesity (64.4%) and did not include women who were overweight; this sample had an average GWG of 12.0±7.2 kg and did not include an intervention. Currently, there are no guidelines for an upper limit related to UPF intake and what factors would influence such a recommendation when tied to a specific health outcome such as GWG. This warrants further investigation related to dietary recommendations of UPF for specific populations and health outcomes.

## Limitations

The current study has limitations. The small sample sizes of the quartiles across different levels of UPF consumption are underpowered to detect differences in GWG below 2.5 kg. Initially, we performed the analyses with the total sample, which were powered to detect differences above 1.9 kg. These analyses also demonstrated no association between PEI-UPF and GWG. As studies in the literature have often used quartiles or quintiles to categorise UPF consumption, we felt that results using quartiles may be more informative to readers. In our sample, the dietary intake profile using the NOVA classification was different than compared to the national average intakes of unprocessed food and UPFs. Moreover, the study used two 24-hour recalls; the best practice around the implementation of the ASA-24 is three recalls at each timepoint to assess usual dietary intake [33]. In addition, social desirability bias may lead to underreporting of UPFs or overreporting of unprocessed food, which could have diluted the association between UPF intake and outcomes. Although some information indicative of food processing level (e.g. place of meals, product brands) was collected, these data are limited and not consistently determined for all food items, which could lead to over or underestimation of UPF. Lastly, we acknowledge these results may not be generalisable to a nationally representative population because of the study design, which included a non-representative sample of women with overweight and obesity. Additionally, the data were collected in the context of an intervention and therefore are not generalisable to free-living conditions.

This study has also strengths. The data came from a randomised and rigorously conducted trial that included state-of-the-art early-life body composition measures. The sample size (n=64) for QMR and ADP had a power of 0.80 to detect a mean difference between quartiles in infant LM and FFM of 10% or above. It is the first study to explore associations between UPF intake and body composition outcomes measured by QMR. Automated coding for the NOVA food categories previously used in publications involving national datasets [30,39] adds to rigor and reproducibility of the current analysis.

## Conclusions

The long-term physiological effects of UPF consumption through the life cycle are unknown. This study investigated the effects of UPF intake on maternal excessive GWG and neonatal FM, FFM and LM measured by two independent methods. While no significant associations between UPF quartiles and GWG or neonatal body composition were observed in the current study, further investigations into the relationships of maternal UPF intake and maternal excessive GWG and neonatal body composition are warranted.

- A previous study has demonstrated a positive association between UPF intake and GWG and neonatal adiposity.
- The current study did not observe an association between UPF intake and GWG nor neonatal body composition variables.
- The current study contributes to a growing body of literature on the effects of UPF consumption across the life cycle.

## Supplementary Information

**Supplementary Table 1:** Maternal characteristics at baseline and GWG by intervention/control group

	Intervention (n=82)	Usual Care (n=74)	Test statistic	P
**Maternal Age (years)**	33.62± 3.96	33.21 ± 4.37	-0.616^a^	0.539
**Gestational Age at Randomisation (weeks)**	14.41 ± .753	14.43 ± .795	0.144^a^	0.886
**Height (cm)**	164.58± 5.43	163.80 ± 6.78	-0.973^a^	0.431
**Weight (kg)**	80.88 ± 12.14	83.24 ± 15.86	1.049^a^	0.296
**Mean BMI Categories (kg/m^2^)**	29.86 ± 3.88	31.0 ± 5.4	1.551^a^	0.117
**>24.9**	54 (65.1%)	41 (55.4%)	1.783^c^	0.182
**>29.9**	29 (34.9%)	33 (44.6%)		
**Parity**			3.042^c^	0.804
**0**	26 (31.7%)	26 (35.1%)		
**1**	25 (30.5%)	25 (33.8%)		
**>2**	31 (37.8%)	23 (31.1%)		
**Race/Ethnicity**			1.208^c^	0.751
**Hispanic**	25 (30.5%)	17 (23.0%)		
**Non-Hispanic African**	16 (19.5%)	16 (21.6%)		
**American**	35 (42.7%)	34 (45.9%)		
**Non-Hispanic White**	6 (7.3%)	7 (9.5%)		
**More than one race**				
**Household Income**			1.967^d^	0.579
**<$24,999**	4 (4.9%)	5 (6.8%)		
**$25,000-$74,9999**	26 (31.7%)	20 (27.0%)		
**>$75,000**	52 (63.4%)	49 (66.2%)		
**College Degree**			1.416^c^	0.291
**No**	17 (20.7%)	10 (13.5%)		
**Yes**	65 (79.3%)	64 (86.5%)		

No between group differences existed at baseline in parent study^18^ a = t statistic reported, b = 2xc chi square reported, c = rx2 chi square reported, d = rx2 Fischers reported

**Supplementary Table 2:** Gestational weight gain and maternal ASA-24 by intervention/control group

	Intervention (n=82)	Usual Care (n=74)	Test statistic	Mean Difference	*P*
	Mean±SD	Mean±SD		Mean±SE	
**Gestational Weight Gain (kg)**	8.04±3.85	9.8±4.4	2.658^a^	1.76±0.66	0.009*
Gestational Weight Gain: adherence to Institute of Medicine guidelines					
Gained Adequately	51 (62.2%)	33 (44.6%)	---		---
Gained Excessively	31 (37.8%)	41 (55.4%)	---		---
Gestational Weight Gain:defined by median (7.4kg)					
Gained Adequately	39(47.6%)	22(29.7%)	---		---
Gained Excessively	43(52.4%)	52(70.3%)	---		---
**Pre-Intervention: Automated Self-Administered 24 Food Recall Week 14**	N=82	N=74			
Total Calories (Kcal)	1817.16±737.4	2088.97 ± 1067.4	1.865^a^	271.8±148.4	0.069
Healthy Eating Index (HEI) 2010 Score (0-100)	56.69 ±14.31	56.09 ±16.32	-0.245^a^	-0.60±2.45	0.807
Total Fruit (0-5)^c^	3.47±1.88	3.39±1.98	2999^b^	-0.78±0.31	0.896
Whole Fruit (0-5)^c^	3.47±2.13	3.12±2.19	3289^b^	-0.35±0.35	0.318
Solid Fats, Alcohol, Added Sugars (SOFAAS) (0-10)^d^	14.06±5.07	13.51±6.01	2511^b^	-0.55±0.89	0.060
**Post-Intervention Automated Self-Administered 24 Food Recall Week 35**	N=82	N=74			
Total calories	1843.4±979.5	1880.7±1072	0.227^a^	37.3±164.3	0.837
**HEI 2010 Score**	62±16	55.8±15.5	-2.465^a^	-6.23±2.52	0.015*
**Total Fruit^c^**	3.83±1.69	3.05±1.96	3735^b^	-0.83±0.29	0.009*
**Whole Fruit^c^**	3.87±2	3.00±2.24	3764^b^	-0.79±0.34	0.004*
**SOFAAS^d^**	14.53±5.67	12.8±5.67	3624^b^	-1.87±0.90	0.036*
**Pre-Intervention NOVA Scores**:					
Group 1	40.0±16.3	36.3±16.7	-1.374 ^a^	-0.036±0.03	0.171
Group 2	4.9±5.5	5.8±8.4	0.551 ^a^	0.006±0.01	0.582
Group 3	10.6±8.9	10.4±11.9	-0.121 ^a^	-0.002±0.02	0.904
Group 4	44.6±18.7	47.8±18.4	1.080 ^a^	0.03±0.03	0.282
**Post-Intervention NOVA Scores**:					
Group 1	40.5±15.8	42.0±17.5	0.569 ^a^	0.151±0.03	0.570
Group 2	5.2±5.9	5.1±5.3	-0.025 ^a^	-0.003±0.01	0.980
Group 3	9.4±8.4	9.7±10.0	0.156 ^a^	0.002±0.01	0.876
Group 4	44.9±18.2	43.1±20.1	-0.562 ^a^	-0.017±0.03	0.575

a = t statistic reported, b =Mann Whitney u distribution reported, c= scored in context of adequacy where the relationship to the HEI total score is positive d= scored in context of moderation where the relationship to HEI total score is negative

**Supplementary Table 3:** Neonatal birth characteristics and body composition by intervention/control group

	Intervention (n = 68)	Usual Care (n = 61)	T-Test Statistic	*p*
Birth weight (g)	3280±421	3160 ±468	-1.465	0.146
Birth length (cm)	49.84±1.983	49.8±2.034	-0.325	0.746
Age at measurement	2.54 ±2.1	2.34±1.8	-0.589	0.557
(days)				
Weight for length z score	-0.22±0.7	-0.43±0.8	-1.57	0.119
**PEAPOD**				
FM (g)	352.5 ±172	329 ±155	-0.806	0.422
FFM (g)	2920±335	2830 ±383	-1.433	0.151
**QMR**				
FM (g)	555±171	535± 164	-0.676	0.500
LM (g)	2360± 262	2260±293	-2.149	0.034*
TBW (g)	2480 ± 282	2390 ± 308	-1.827	0.070

FM: Fat mass; FFM: Fat-free mass; LM: Lean mass TBW: Total body water

**Supplementary Table 4:** Maternal covariates and neonatal birth characteristics by gestational weight gain (above or below the mode)

Maternal^+^	GWG N=156		Mean Difference ±SE	*p*
	<7.4kg (n=61)	>7.4kg (n=95)		
**Gestational Age (week) (mean)^a^**	14.5±0.70	14.35±0.80	0.194±.13	0.06
**Maternal (years) (mean)Age ^a^**	33.6±4.5	33.3±4.0	0.257±.68	0.35
**HEI Avg (0-100)**	54.3.1±10.4	59.9±13.5	5.63+2.0	<0.01*
Parity (%)^b^			1.56	0.46
**1**	28	37		
**2**	33	32		
**3+**	39	31		
Race (%)^b^			10.87	0.001*
**White**	28	55		
**Non-white**	78	45		
Income (%)^b^			0.03	0.865
≤**$75,000**	36	35		
**>$75,000**	64	65		
College (%)			3.71	0.05*
**No**	25	13		
**Yes**	75	87		
Group (%)^b^			5.19	0.02*
**UC**	36	55		
**LI**	64	45		
NOVA PEI				
**G1**	39.7±15	42.3±.17	2.4±3.0	0.37
**G2**	5.3±5.7	5.1±6.0	0.1±.0.09	0.43
**G3**	8.0±8.0	10.0±10.0	2.4±1.0	0.06
**G4**	46.9±17.6	42.2±19.8	4.7±3.1	0.07
**GWG**	4.87±1.9	11.44±3.1	6.57±0.44	0.001**
**Obesity baseline) (at (%)^b^**			29.5	0.000*
**BMI <29.9 kg/m^2^**	34	78		
**BMI >29.9 kg/m^2^**	66	22		

a = t statistic reported, b =Mann Whitney u distribution reported, c= scored in context of adequacy where the relationship to the HEI total score is positive d= scored in context of moderation where the relationship to HEI total score is negative G1 = unprocessed foods, G2= minimally processed culinary ingredients, G3= processed foods, G4= ultra-processed foods; %= contribution to total energy intake;

**Supplementary Table 5:** NOVA group energy contribution and diet quality differences between week 14 and 35 according to PEI-UPF quartiles^a^

	Q1 (0-36.09%) N=39 (Mean±SD)		Q2 (36.10-45.23%) N=39 (Mean±SD)		Q3 (45.24-54.73%) N=39 (Mean±SD)		Q4 (54.74-75.25%) N=39 (Mean±SD)		Total N=156 Mean±SD	
	Mean Week 14	Mean Week 35	Mean Week 14	Mean Week 35	Mean Week 14	Mean Week 35	Mean Week 14	Mean Week 35	Mean Week 14	Mean Week 35
**G1 %**	48.5±14.9	54.6±13.2	41.1±17.4	44.6±17.8	37.5±13.0	34.8±10.4	25.9±12.4	30.8±13.2	38.2 ±16.6	41.2±16.6
**G2 %**	5.6±6.2	7.5±7.2	7.3±9.9	4.1±4.4*	5.1±6.4	4.6±4.4	2.6±3.2	4.6±5.8*	5.1±7.0	5.2±5.7
**G3 %**	14.8±14.1	11.4±9.9	9.4±8.5	12.1±10.6	10.8±9.8	7.9±9.3	6.9±6.4	6.8±5.3	10.5±10.4	9.5±9.2
**G4 %**	31.1±14.2	26.6±15.3	42.2±14.2	39.2±13.7	46.5±12.9	52.7±13.1	64.6±15.6	57.7±16.8	46.1±18.6	44.0±19.0
**Total**	2004.7±	1747.1±	1881.4±	1845.9±	2042.1±	1863.9±	1856.2±	1987.4±	1946 ±	1861 ±1
**Kcals**	927.2	918.9	975.9	1440.9	962.9	806.2	811.7	808.4	916	021
**HEI (0-100)**	62.1±13.4	63.2±17.1	58.3±18.4	60.2±18.6	55.9±10.9	56.3±12.7	49.3±15.0	56.4±14.7*	56.4±15.3	59.0±16.0

G1 = unprocessed foods, G2= minimally processed culinary ingredients, G3= processed foods, G4= ultra-processed foods; ^a^ Maternal Quartiles (mean week 14 and 35) (n=156) (mean, min-max range): Q1 =28.8 (12.8-36.09%), Q2=40.7 (36.10-45.23%), Q3=49.6 (45.24-54.73%), Q4=61.2 (54.74-75.25%); %= contribution to total energy intake; Kcal=kilocalorie; HEI=healthy eating index; *Statistically significant mean difference between week 14 and 35: p<0.05 (t-test)

## References

[1] Pi-Sunyer X. (2009). The medical risks of obesity. Postgrad Med.

[2] Tanentsapf I, Heitmann BL, Adegboye AR (2011). Systematic review of clinical trials on dietary interventions to prevent excessive weight gain during pregnancy among normal weight, overweight and obese women. BMC Pregnancy Childbirth.

[3] Lenoir-Wijnkoop I, van der Beek EM, Garssen J, Nuijten MJ, Uauy RD (2015). Health economic modeling to assess short-term costs of maternal overweight, gestational diabetes, and related macrosomia - a pilot evaluation. Front Pharmacol..

[4] Clifton RG, Evans M, Cahill AG, Franks PW, Gallagher D, Phelan S (2016). LIFE-Moms Research Group. Design of lifestyle intervention trials to prevent excessive gestational weight gain in women with overweight or obesity. Obesity (Silver Spring).

[5] Barker DJP (2012). Sir Richard Doll Lecture. Developmental origins of chronic disease. Public Health.

[6] Brenseke B, Prater MR, Bahamonde J, Gutierrez JC (2013). Current thoughts on maternal nutrition and fetal programming of the metabolic syndrome. J Pregnancy.

[7] Almond D, Currie J (2011). Killing me softly: the fetal origins hypothesis. J Econ Perspect.

[8] Norman JE, Reynolds RM (2011). The consequences of obesity and excess weight gain in pregnancy. Proc Nutr Soc.

[9] Metzger BE, Lowe LP, Dyer AR, Trimble ER, Sheridan B, Hod M (2009). Hyperglycemia and Adverse Pregnancy Outcome (HAPO) Study: associations with neonatal anthropometrics. Diabetes.

[10] Shapiro AL, Kaar JL, Crume TL, Starling AP, Siega-Riz AM, Ringham BM (2016). Maternal diet quality in pregnancy and neonatal adiposity: the Healthy Start Study. Int J Obes (Lond).

[11] Horan MK, McGowan CA, Gibney ER, Donnelly JM, McAuliffe FM (2014). Maternal low glycaemic index diet, fat intake and postprandial glucose influences neonatal adiposity--secondary analysis from the ROLO study. Nutr J.

[12] Kizirian NV, Markovic TP, Muirhead R, Brodie S, Garnett SP, Louie JC (2016). Macronutrient balance and dietary glycemic index in pregnancy predict neonatal body composition. Nutrients.

[13] Shapiro ALB, Ringham BM, Glueck DH, Norris JM, Barbour LA, Friedman JE (2017). Infant adiposity is independently associated with a maternal high fat diet but not related to niacin intake: the Healthy Start study. Matern Child Health J.

[14] Horan MK, McGowan CA, Gibney ER, Donnelly JM, McAuliffe FM (2015). The association between maternal dietary micronutrient intake and neonatal anthropometry–secondary analysis from the ROLO study. Nutr J.

[15] Dodd JM, Crowther CA, Robinson JS (2008). Dietary and lifestyle interventions to limit weight gain during pregnancy for obese or overweight women: a systematic review. Acta Obstet Gynecol Scand.

[16] Asbee SM, Jenkins TR, Butler JR, White J, Elliot M, Rutledge A (2009). Preventing excessive weight gain during pregnancy through dietary and lifestyle counseling: a randomized controlled trial. Obstet Gynecol.

[17] Polley BA, Wing RR, Sims CJ (2002). Randomized controlled trial to prevent excessive weight gain in pregnant women. Int J Obes Relat Metab Disord.

[18] Gallagher D, Rosenn B, Toro-Ramos T, Paley C, Gidwani S, Horowitz M (2018). Greater neonatal fat-free mass and similar fat mass following a randomized trial to control excess gestational weight gain. Obesity (Silver Spring).

[19] Vesco KK, Karanja N, King JC, Gillman MW, Leo MC, Perrin N (2014). Efficacy of a group-based dietary intervention for limiting gestational weight gain among obese women: a randomized trial. Obesity (Silver Spring).

[20] Martínez Steele E, Popkin BM, Swinburn B, Monteiro CA (2017). The share of ultra-processed foods and the overall nutritional quality of diets in the US: evidence from a nationally representative cross-sectional study. Popul Health Metr..

[21] Mendonça RD, Lopes AC, Pimenta AM, Gea A, Martinez-Gonzalez MA, Bes-Rastrollo M (2017). Ultra-processed food consumption and the incidence of hypertension in a Mediterranean cohort: the Seguimiento Universidad de Navarra Project. Am J Hypertens.

[22] Rauber F, Campagnolo PD, Hoffman DJ, Vitolo MR (2015). Consumption of ultra-processed food products and its effects on children's lipid profiles: a longitudinal study. Nutr Metab Cardiovasc Dis.

[23] Fiolet T, Srour B, Sellem L, Kesse-Guyot E, Allès B, Méjean C (2018). Consumption of ultra-processed foods and cancer risk: results from NutriNet-Santé prospective cohort. BMJ.

[24] Beslay M, Srour B, Méjean C, Allès B, Fiolet T, Debras C (2020). Ultra-processed food intake in association with BMI change and risk of overweight and obesity: a prospective analysis of the French NutriNet-Santé cohort. PLoS Med.

[25] Srour B, Fezeu LK, Kesse-Guyot E, Allès B, Méjean C, Andrianasolo RM (2019). Ultra-processed food intake and risk of cardiovascular disease: prospective cohort study (NutriNet-Santé). BMJ.

[26] Srour B, Fezeu LK, Kesse-Guyot E, Allès B, Debras C, Druesne-Pecollo N (2020). Ultraprocessed food consumption and risk of type 2 diabetes among participants of the NutriNet-Santé Prospective Cohort. JAMA Intern Med.

[27] Martínez Steele E, Juul F, Neri D, Rauber F, Monteiro CA (2019). Dietary share of ultra-processed foods and metabolic syndrome in the US adult population. Prev Med.

[28] Monteiro CA, Cannon G, Levy R, Moubarac J-C, Jaime P, Martins AP (2016). NOVA. The star shines bright. [Food classification. Public Health]. World Nutr..

[29] Poti JM, Braga B, Qin B (2017). Ultra-processed food intake and obesity: what really matters for health-processing or nutrient content?. Curr Obes Rep.

[30] Rohatgi KW, Tinius RA, Cade WT, Steele EM, Cahill AG, Parra DC (2017). Relationships between consumption of ultra-processed foods, gestational weight gain and neonatal outcomes in a sample of US pregnant women. PeerJ.

[31] Peaceman AM, Clifton RG, Phelan S, Gallagher D, Evans M, Redman LM (2018). LIFE‐ Moms Research Group. Lifestyle interventions limit gestational weight gain in women with overweight or obesity: LIFE-Moms prospective meta-analysis. Obesity (Silver Spring)..

[32] USDA ARS. Food and Nutrient Database for Dietary Studies..

[33] Guenther PM, Kirkpatrick SI, Reedy J, Krebs-Smith SM, Buckman DW, Dodd KW (2014). The Healthy Eating Index-2010 is a valid and reliable measure of diet quality according to the 2010 Dietary Guidelines for Americans. J Nutr..

[34] Urlando A, Dempster P, Aitkens S (2003). A new air displacement plethysmograph for the measurement of body composition in infants. Pediatr Res.

[35] Deierlein AL, Thornton J, Hull H, Paley C, Gallagher D (2012). An anthropometric model to estimate neonatal fat mass using air displacement plethysmography. Nutr Metab (Lond).

[36] Andres A, Gomez-Acevedo H, Badger TM (2011). Quantitative nuclear magnetic resonance to measure fat mass in infants and children. Obesity (Silver Spring).

[37] Toro-Ramos T, Paley C, Wong WW, Pi-Sunyer FX, Yu WW, Thornton J (2017). Reliability of the EchoMRI Infants System for water and fat measurements in newborns. Obesity (Silver Spring).

[38] Hall KD, Ayuketah A, Brychta R, Cai H, Cassimatis T, Chen KY (2019). Ultra-processed diets cause excess calorie intake and weight gain: an inpatient randomized controlled trial of ad libitum food intake. Cell Metab.

[39] Martínez Steele E, Baraldi LG, Louzada ML, Moubarac JC, Mozaffarian D, Monteiro CA (2016). Ultra-processed foods and added sugars in the US diet: evidence from a nationally representative cross-sectional study. BMJ Open.

